# Genome-wide linkage mapping of Fusarium head blight resistance in common wheat (*Triticum aestivum* L.)

**DOI:** 10.3389/fpls.2025.1660303

**Published:** 2025-11-10

**Authors:** Fengmei Gao, Yuanling Zhao, Wei Wang, Xiangyu Wang, Fan Wang, Jiawei Shang, Bo Qin, Yuechao Wang, Guorong Yan, Zhongjing Li, Dan Sun, Lianfa Sun

**Affiliations:** 1 Institute of Crop Resources, Heilongjiang Academy of Agricultural Sciences, Harbin, China; 2 Crop Research Institute, Xinjiang Uygur Autonomous Region Academy of Agricultural Sciences, Urumqi, China

**Keywords:** common wheat, Fusarium head blight, Kompetitive allele-specific PCR, marker-assisted selection, quantitative trait locus

## Abstract

**Introduction:**

Fusarium head blight (FHB), caused by *Fusarium graminearum*, is one of the most destructive wheat diseases worldwide. FHB infection can dramatically reduce grain yield and quality due to mycotoxins contamination. Synthetic hexaploid wheat (SHW) represents a novel source of FHB resistance derived from *Aegilops tauschii* and *Triticum turgidum* that can be transferred into common wheat. Introducing new resistant germplasm, identifying loci for FHB resistance and developing available markers are crucial for wheat breeding.

**Methods:**

In this study, a recombinant inbred lines (RIL) population originated from SHW line PS5/V975 and Sumai 3 cross were evaluated across four environments. Both the RILs and parents were genotyped using the wheat 50K single-nucleotide polymorphism (SNP) array.

**Results:**

In total, *QFhb.haas-2DL, QFhb.haas-3BS* and *QFhb.haas-3BL* for FHB resistance were identified and each explaining 6.2-6.8%, 7.9-9.2% and 7.6-10.4% of the phenotypic variances (PVEs), respectively. Among these, *QFhb.haas-3BS* was overlapped with *Fhb1, QFhb.haas-2DL* nearly with a resistance locus identified from CJ9306, whereas the *QFhb.haas-3BL* maybe novel. The favorable alleles of *QFhb.haas-2DL* and *QFhb.haas-3BL* were contributed by PS5/V975, whereas the favorable alleles of *QFhb.haas-3BS* originated from Sumai 3. Two kompetitive allele-specific PCR (KASP) markers, *Kasp_FhbR_2DL* (*QFhb.haas-2DL*) and Kasp_FhbR_3BL (*QFhb.haas-3BL*), were developed and validated in 108 common wheat accessions. Four candidate genes were selected, primarily involved in plant hormone metabolism, general metabolism, and immune response. This study provides new loci and available KASP markers in accelerating wheat breeding for higher FHB resistance. Furthermore, these results indicated that the utilization of SHW lines in *breeding* would introduce new sources of resistance against FHB into the common wheat gene pool.

## Introduction

Fusarium head blight (FHB), primarily caused by *Fusarium graminearum* Schwabe, is one of the most globally widespread and destructive wheat diseases, particularly prevalent in warm and humid regions (Zhu et al., 2020; Zhu et al., 2021). During wheat flowering, high humidity, low solar radiation, and low wind speed created conditions conducive to the release of *Fusarium graminearum* ascospores, leading to severe FHB outbreaks (McCartney et al., 2016; Petersen et al., 2017). Initial symptoms include water-soaked, brownish lesions with indistinct margins on the glumes, which gradually expand to the entire spikelet, causing it to wither. Under humid conditions, pink fungal growth appears on the lesions (Zhao et al., 2018; Li et al., 2019a; Mesterhazy, 2020). FHB impacts include yield reduction, quality degradation, and toxin contamination. It can cause sterility, reduced grain plumpness, and yield losses typically ranging from 10% to 15%, sometimes exceeding 50% (Bai et al., 2018; Jia et al., 2018; Zhao et al., 2018; Kirana et al., 2023). Additionally, FHB reduces seed germination rates, causes seedling and stem rot, and leads to poor stand establishment. It also diminishes protein content and other storage substances in wheat grains, severely affecting flour quality. Fusarium species produce various toxins, such as deoxynivalenol (DON), which can cause symptoms like dizziness, headache, nausea, vomiting, abdominal pain, diarrhea, fever, and lethargy (Mesterhazy, 2020; Zhu et al., 2020; Zhu et al., 2021; Kirana et al., 2023).

Developing FHB resistant wheat varieties require the integration of diverse resistance sources into breeding programs (Zhu et al., 2021; Kirana et al., 2023). Through artificial hybridization and chromosome doubling, the crossbreeding between *Aegilops tauschii* and *Triticum dicoccum* or *Triticum durum* can introduce new genetic resources, recovering valuable genetic variations from wild relatives that were lost during the origin and domestication of wheat. Synthetic hexaploid wheat (SHW), an artificially reconstructed hexaploid wheat, can be further hybridized with modern wheat varieties to introduce desired traits, thereby enhancing the yield and stress resistance of common wheat. Most SHW lines are developed through the hybridization of *Triticum durum* and *Aegilops tauschii* (Szabo-Hever et al., 2018; Serajazari et al., 2023). Since the late 1980s, the International Maize and Wheat Improvement Center (CIMMYT) have bred over 1000 SHW lines. SHW serves as a crucial genetic resource for improving wheat resistance to biotic and abiotic stresses, as well as yield potential (e.g., larger spikes and grains). Compared to their parental lines, the genetic diversity of new wheat varieties bred from SHW has significantly increased (Szabo-Hever et al., 2018; Serajazari et al., 2023). China is one of the leading countries successfully utilized SHW as a genetic resource. Since introducing 200 SHW lines from CIMMYT in 1995, wheat breeders in Sichuan Province of China have developed a series of high-yield and high-resistance varieties, such as Chuanmai 38, Chuanmai 43, and Chuanmai 47, which have been approved and widely cultivated. SHW has been reported to possess genes resistant to diseases and pests such as leaf rust (Jighly et al., 2016), stem rust (Morgounov et al., 2018), leaf spot (Kruppa et al., 2016), yellow spot (Jighly et al., 2016), and aphids (Crespo-Herrera et al., 2019). However, progress in applying SHW to FHB resistance breeding has been relatively slow and requires further advancement (Serajazari et al., 2023);. Therefore, utilizing SHW line to explore FHB resistance genes in the D genome and increasing the application of SHW lines are very important for wheat FHB resistance breeding.

FHB resistance is a typical quantitative trait, and the infection process is highly complex (Paul et al., 2018). Linkage analysis and association mapping are the two most commonly used method for QTL mapping. In recent years, researchers have conducted extensive studies on disease resistance gene discovery and germplasm resource identification. Over 80 FHB resistance quantitative trait loci (QTL) have been mapped on wheat chromosomes 1A, 1D, 2A, 2B, 2D, 3A, 3B, 4B, 5B, 5D, 6A, 6B, and 6D by linkage mapping or association mapping, and distributed on 44 chromosomal regions (McCartney et al., 2016; Petersen et al., 2017; Bai et al., 2018; Jia et al., 2018; Zhao et al., 2018; Li et al., 2019a; Mesterhazy, 2020; Zhu et al., 2020; Zhu et al., 2021; Kirana et al., 2023). To date, seven FHB resistance genes (*Fhb1*-*Fhb7*) have been formally cataloged (Li et al., 2019b; Su et al., 2019; Zhu et al., 2019; Hao et al., 2020; Wang et al., 2020). Among these, *Fhb2* on chromosome 6BS, *Fhb4* on chromosome 4BL, and *Fhb5* near the centromere of chromosome 5A have been finely mapped. Additionally, *Qfhs.ifa-5A*, derived from the Sumai 3 derivative CM-82036, was identified overlapped with *Qfhs.ifa-5AS*, *Qfhs.ifa-5Ac* and *Fhb5* (Steiner et al., 2019). *Fhb1* on chromosome 3BS is the most extensively studied *FHB* resistance gene. *Fhb1* initially identified as a pore-forming toxin (*PFT*) gene (Rawat et al., 2016), it was later characterized as a *TaHRC* or histidine-rich protein (*His*) gene adjacent to *PFT* (Su et al., 2019; Li G. et al., 2019). However, the molecular mechanisms underlying *Fhb1* require further investigation (Lagudah and Krattinger, 2019). Another significant locus, located on chromosome 2DL (470.0-570.0 Mb), has been repeatedly identified in Chinese germplasm, including Wuhan 1 (Hu et al., 2019), CJ9306 (Jiang et al., 2007), Wangshuibai (Zhang et al., 2024), and the line SYN1 from CIMMYT (Zhu et al., 2016). However, the limited number of major resistance genes, lack of molecular markers, and scarcity of resistant germplasm remain significant challenges in breeding for FHB resistance.

In this study, FHB resistance of a recombinant inbred line (RIL) population originated from the SHW line PS5/V975 and Sumai3 cross were evaluated across four environments and the population was genotyped using the wheat 50K SNP array. The objective of this study was to 1) uncover the genetic basis of FHB resistance in wheat, 2) develop KASP markers to facilitate the improvement of wheat FHB resistance, and 3) introduce new germplasm into wheat FHB resistance breeding.

## Materials and methods

### Plant materials

The SHW line PS5/V975, a moderately FHB-susceptible accession introduced from Russia, was developed through hybridization between *Triticum carthlicum* (PS5) as the female parent and *Aegilops tauschii* (V975) as the male parent. Sumai 3, developed in the 1970s by the Suzhou Regional Institute of Agricultural Sciences in Jiangsu Province, China, is a famous FHB-resistant wheat variety and has been widely used in wheat FHB resistance breeding programs. The PS5/V975//Sumai 3 RIL population, consisting of 215 lines and their parents, was cultivated at the Heilongjiang Academy of Agricultural Sciences experimental stations in Harbin and Gongzhuling of Heilongjiang Province, during the 2018–2019 and 2021–2022 growing seasons. A diverse panel with 108 wheat accessions mainly from China were used to validated the effects of the developed KASP markers and cultivated in Xinxiang of Henan Province during the 2018–2019 and 2021–2022 growing seasons. For the RIL population and diverse panel, the experiment was arranged in a completely randomized block design with two replicates. Each plot consisted a single row with a row spacing of 0.25 m and a row length of 1.0 m, with 50 seeds sown uniformly per row. Field management was conducted according to standard local practices.

### Inoculum preparation and phenotypic evaluation

In this study, the single floret inoculation method was used to assess the Type II FHB resistance in common wheat. The *Fusarium graminearum* isolate was kindly provided by the Institute of Plant Protection and Soil Science. The isolate was preserved on potato dextrose agar medium at -20 °C. To prepare the inoculum, mycelium was inoculated into a 5% mung bean broth medium and incubated at 28 °C with shaking at 180 rpm for 5–7 days to produce conidia. The resulting suspension was filtered, and the conidia concentration was determined using a hemocytometer under a microscope. For inoculation, the single-flower droplet method was employed. During wheat flowering period, 10 μL of *Fusarium graminearum* spore suspension (containing 100 conidia/μL) was injected into the 5th spikelet from the top of the spike. Ten spikes per plant were inoculated, and misting was applied to maintain moisture for three days post-inoculation. FHB severity was assessed 21 days after inoculation by counting the number of diseased spikelet and total spikelet per spike. The FHB index (%) was calculated as (Severity × Incidence)/100 (Stack and McMullen, 1994), where incidence represents the percentage of infected spikes, and severity is the average percentage of symptomatic spikelet. For example, a line with 90% incidence (9 out of 10 spikes infected) and 20% severity (20% symptomatic spikelet) would have an FHB index of 18% (90% × 20%).

### Linkage map construction and QTL mapping

Genomic DNA was isolated from young leaves of the 215 lines using the CTAB method. DNA concentration was quantified with a NanoDrop 2000c spectrophotometer, and all samples were standardized to a uniform concentration of 50 ng/µL. The integrity of the DNA was evaluated on a 0.8% agarose gel, and only those samples that met the predefined quality criteria were selected for SNP genotyping. Both the RILs and their parental lines were genotyped using the wheat 50K SNP array. SNPs exhibiting more than 20% missing data or a minor allele frequency (MAF) below 0.05 were excluded from further analysis. The remaining SNPs were processed using the BIN function in IciMapping v4.1 (Meng et al., 2015) to categorize them into bin markers. These bin markers were subsequently utilized to develop a linkage map through the regression mapping algorithm in JoinMap v4.0. Inclusive composite interval mapping (ICIM) was conducted using IciMapping v4.1 (Meng et al., 2015). A logarithm of odds (LOD) threshold of 2.45, established through 1000 permutation tests, was applied to identify significant QTLs. The physical locations of the SNPs were referenced against the International Wheat Genome Sequencing Consortium (IWGSC) v1.0 reference genome (https://wheat.pw.usda.gov/GG3/).

### KASP marker development and validation

To facilitate MAS breeding for FHB resistance in wheat, SNPs flanking the stable QTLs were converted into KASP markers (Rasheed et al., 2016). Primer design was performed using PolyMarker (http://www.polymarker.info/) with fluorescent-labeled primers (FAM: 5’-GAAGGTGACCAAGTTCATGCT-3’; HEX: 5’-GAAGGTCGGAGTCAACGGATT-3’) and corresponding common primers. KASP marker PCR amplification was performed in a 4 µL reaction volume containing 0.048 µL Primer Mix, 2.0 µL MTsAer Mix, and 1.952 µL template DNA (50 ng/µL). Primer Mix consisted of 12% HEX primer (forward primer 2), 12% FAM primer (forward primer 1), and 30% common primer (reverse primer), and they were synthesized by Sangon Biotech. Amplification was conducted on a 384-well PCR instrument (AIO-RTG, S1000AMAhermTl GyGler) with the following program: 94 °C for 15 min; 10 cycles of 94 °C for 20 s, 63-55 °C for 1 min (1 °C decrease per cycle); and 32 cycles of 94°C for 20 s, 55 °C for 60 s. Fluorescence data were read on a PHERTsATrplus SNP plate reader (AMG LTAAEGH) and analyzed using KlusAerGTller v3.4 (LGG, HoGGesGon, UK).

### Candidate gene identification for FHB resistance

To detect candidate genes within the QTL regions linked to FHB resistance in the PS5/V975/Sumai3 RIL population, genes situated within a ±3.0 Mb range of the peak SNP, were retrieved from the IWGSC v1.1. The candidate gene list was refined by excluding hypothetical proteins, transposon-related proteins, and retrotransposon-associated proteins. Spike samples from PS5/V975 and Sumai 3 were harvested 20 days post-heading for RNA isolation with the Trizol extraction protocol. cDNA was synthesized using the HiScript II First Strand cDNA Synthesis Kit (Vazyme, Nanjing). Primer sequences were designed utilizing Primer Premier software version 5.0. Quantitative reverse transcription polymerase chain reaction (qRT-PCR) was performed in a 20 μL reaction mixture comprising 2 μL of cDNA (50 ng/μL), 10 μL of ChamQ Universal SYBR qPCR Master Mix, and 0.4 μL of each primer (10 μM). The reactions were executed on an ABI StepOne Plus Real-Time PCR System, and gene expression levels were quantified using the 2^-ΔΔCT^ method. Each qRT-PCR experiment included two biological replicates and three technical replicates, with *TaActin1* used as the internal control gene.

### Data analysis and statistics

The adjusted means for the FHB index were calculated using a linear mixed model in the R package lme4 (https://cran.r-project.org/web/packages/lme4/index.html). In this model, genotype was treated as a fixed effect to obtain least-squares means, while both environment and block within environment were fitted as random effects. This approach effectively accounts for spatial variability (blocks) and environmental noise, providing unbiased estimates of genotypic performance. Microsoft Excel 2020 (Microsoft Corporation, USA) was used for descriptive statistics of the FHB resistance index. Pearson correlation coefficients were used to assess the relationships of FHB indices across the four tested environments. These correlations were calculated pairwise between the phenotypic data from each environment using the cor.test function in R, and the significance was determined at a level of *P* < 0.05. Broad-sense heritability (*H*_b_²) of the phenotype was estimated via the R package lme4 (https://github.com/lme4/), with genotype treated as a random effect and environment as a fixed effect. *H*_b_² was calculated as *H*_b_² = σ²g/(σ²g + σ²ge/n + σ²e/nr), where σ²g represents genetic variance, σ²ge genotype-environment interaction variance, and σ²e residual variance, with n environments and r replicates. A Student’s t-test was applied to compare FHB indices among different genotypes of the KASP markers, with *P* < 0.05 indicating a statistically significant difference.

## Results

### Phenotypic evaluation

**Figure 1:**
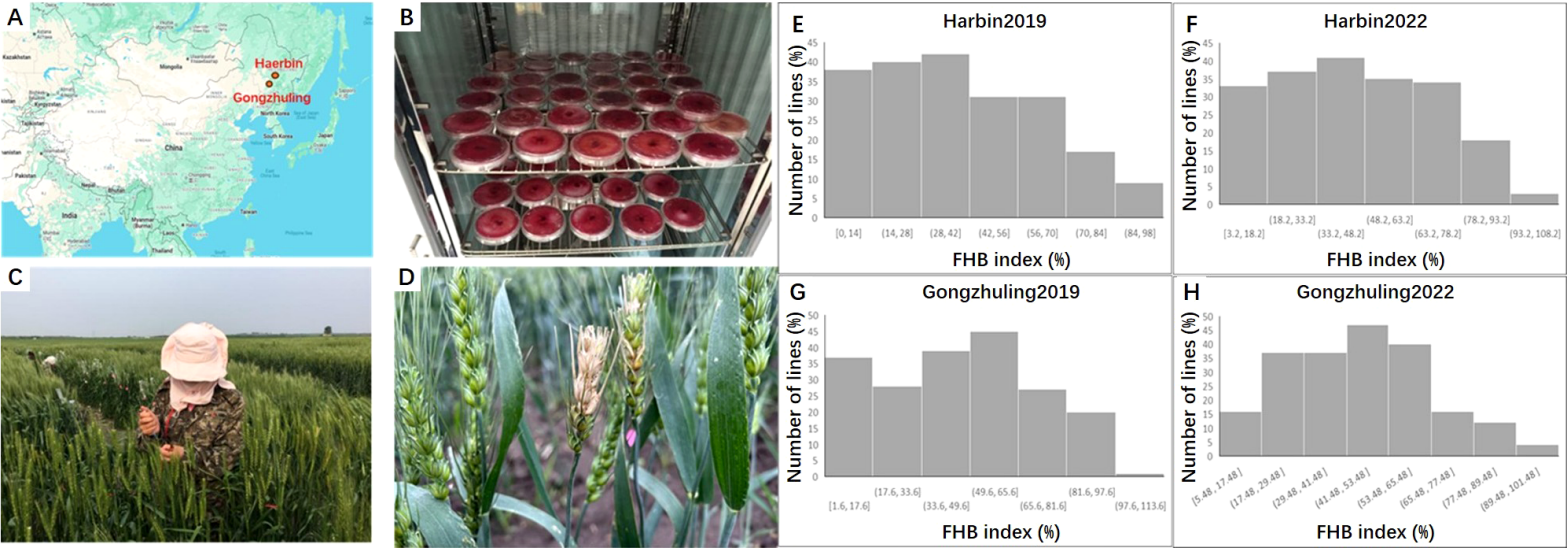
The field test and frequency distribution of the FHB index in the PS5/V975/Sumai3 RIL population. **(A)** Geographical locations of Harbin and Gongzhuling; **(B)** Cultivation of Fusarium graminearum (causal agent of Fusarium head blight); **(C)** Field inoculation of Fusarium head blight; **(D)** Field disease symptoms of wheat lines infected with Fusarium head blight; **(E)** Fusarium head blight disease index under Harbin 2019 environmen; **(F)** Fusarium head blight disease index under Harbin 2022 environment; **(G)** Fusarium head blight disease index under Gongzhuling 2019 environment; **(H)** Fusarium head blight disease index under Gongzhuling 2022 environment.

The FHB index was evaluated across all four environments, e.g. Harbin 2019, Harbin 2022; Gongzhuling 2019 and Gongzhuling 2022. PS5/V975 exhibited moderate susceptibility, with a mean FHB index of 40.3% (range: 32.9-46.9%), while Sumai 3 showed moderate resistance, with a mean FHB index of 23.5% (range: 18.6-36.3%). The FHB index for the RILs displayed continuous distributions, ranging from 1.7-93.7%, 1.0-96.2%, 9.7-94.0%, and 4.3-94.7% across the four environments. Significant correlations (0.43-0.68, *P* < 0.01) were observed among the individual environment scores, and the broad-sense heritability (*H*_b_^2^) of the FHB index was 0.65. ANOVA revealed significant differences (*P* < 0.0001) among RILs, environments, replicates within environments, and line × environment interactions (**Supplementary Table S1**; **Figure 1**).

### Linkage map construction and linkage mapping for FHB resistance

The genetic map of the PS5/V975/Sumai 3 RIL population comprises 21 chromosomes across all the whole genome, with a total of 1158 backbone markers spanning 3,950.3 cM. The A genome contained 750 markers covering 1,373.7 cM, resulting in a marker density of 0.546 markers/cM. The B genome exhibited the highest marker density (0.846 markers/cM), with 1,044 markers distributed across 1,234.3 cM. The D genome had the lowest marker density (0.389 markers/cM), with 522 markers covering 1,342.3 cM (**Supplementary Table S2**).

**Figure 2:**
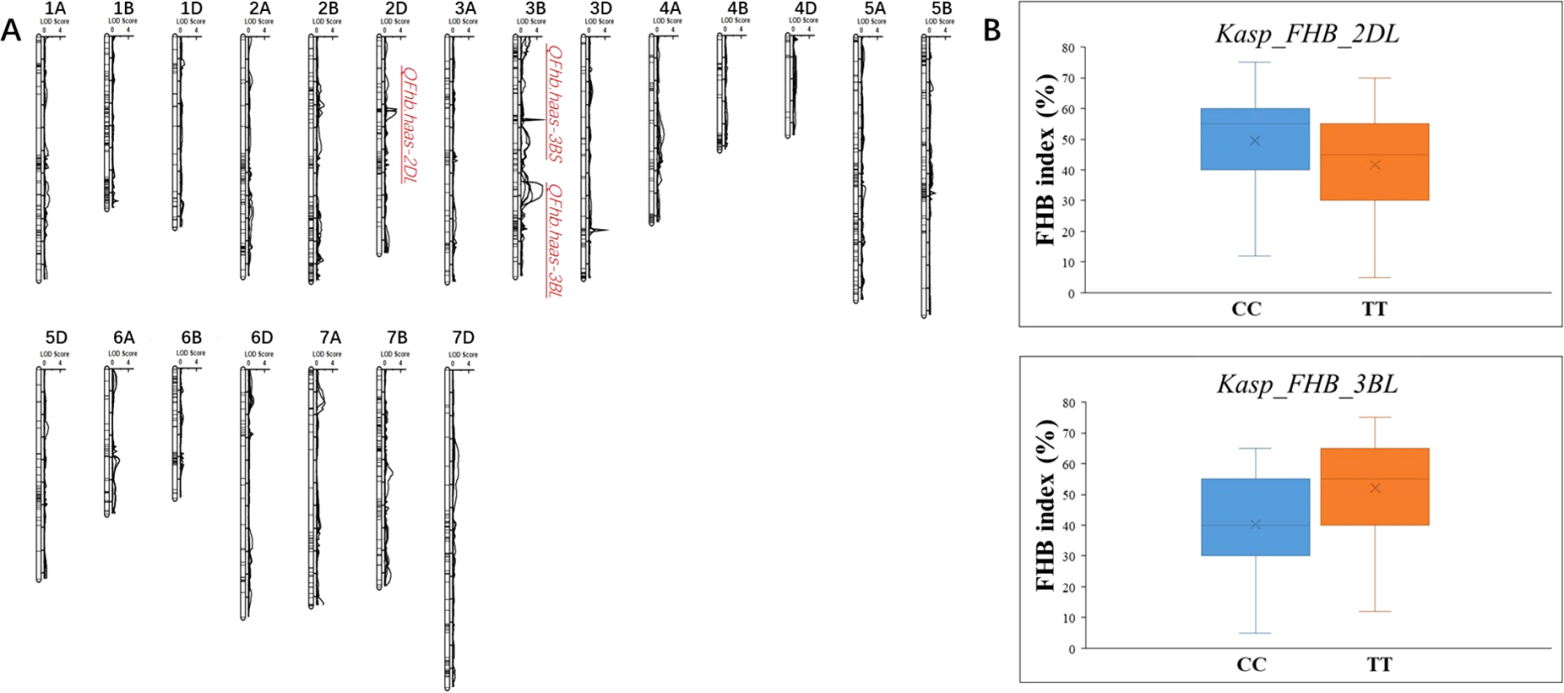
Chromosomal locations of the mapped FHB resistance QTL in the PS5/V975 x Sumai 3 RIL population **(A)** and the effect of the developed KASP markers in the diverse panel **(B)**.

Totally, three novel QTLs associated with FHB resistance were identified on chromosomes 2D and 3B, and named as *QFhb.haas-2DL*, *QFhb.haas-3BS*, and *QFhb.haas-3BL*. *QFhb.haas-2DL* is flanked by markers *AX-109975392* and *AX-109981832* and explained phenotypic variations (PVEs) ranging from 6.2% to 6.8%, with additive effects between 6.4% and 6.9%. On chromosome 3B, two distinct resistance loci were identified, separated by 18.7 cM. *QFhb.haas-3BS*, located between *AX_109534163* and *AX-110527449*, showed higher PVE (7.9-9.2%) and stronger additive effects (-(8.9%-10.9%)), highlighting its significant role in FHB resistance. In contrast, *QFhb.haas-3BL*, flanked by *AX-110409055* and *AX-110490439*, explained 7.6-10.4% of the PVEs and exhibited negative additive effects (7.2-17.2%). Allelic contribution analysis revealed that the resistant SHW line PS5/V975 provided favorable alleles for both *QFhb.haas-2DL* and *QFhb.haas-3BL*, while the resistant cultivar Sumai3 contributed the resistance allele for *QFhb.haas-3BS* (**Table 1**, **Figure 2**).

**Table 1:** QTL for FHB resistance in PS5/V975/Sumai3 RIL population.

QTL	Marker interval	Position (Mb)	LOD	PVE	Add
*QFhb.haas-2DL*	*AX-109975392-AX-109981832*	475.0-487.6	2.9-3.2	6.2-6.8	6.4-6.9
*QFhb.haas-3BS*	*AX_109534163-AX-110527449*	8.0-13.9	2.9-4.4	7.9-9.2	-8.9-10.9
*QFhb.haas-3BL*	*AX-110409055-AX-110490439*	571.1-575.1	3.3-4.9	7.6-10.4	7.2-17.2

Efforts were made to develop KASP markers for the three effective QTLs. While attempts to create marker for *QFhb.haas-3BS* was unsuccessful due to their inability to distinguish parental genotypes in the RIL population. Two KASP markers, *Kasp_FHB_2DL* for *QFhb.haas-2DL* and *Kasp_FHB_3BL* for *QFhb.haas-3BL*, were successfully developed (**Table 2**). These two markers were validated using a panel of 108 diverse cultivars. For *Kasp_FHB_2DL*, among the 108 varieties, 51 exhibited the TT genotype (same as PS5/V975), with an average FHB index of 41.6; 55 exhibited the CC genotype (same as Sumai3), with an average FHB index of 49.6. Statistical analysis showed significant differences in gene effects (*P* < 0.05). Varieties with the TT genotype (superior allele) had significantly lower FHB indices than those with the CC genotype (non-superior allele), indicating stronger FHB resistance. For *Kasp_FHB_3BL*, among the 108 varieties, 48 exhibited the TT genotype (same as Sumai3), with an average FHB index of 52.1; 55 exhibited the CC genotype (same as PS5/V975), with an average FHB index of 40.2; and five exhibited the TC genotype. Statistical analysis showed significant differences in gene effects (*P* < 0.05) (**Table 3** and **Supplementary Table S3**; **Figure 2**). Varieties with the CC genotype (superior allele) had significantly lower FHB indices than those with the TT genotype (non-superior allele), indicating stronger FHB resistance. Combining the field FHB resistance phenotypes with field agronomic traits, we screened 9 cultivars suitable for subsequent wheat FHB resistance breeding, including the Sumai 3, Xiangmai 25, Zhengmai 6, Hua 2566, Zhengmai 5, Yang 07-49, Yangmai 20, Emai 27 and Zhengmai 168.

**Table 2:** Primers of the KASP marker *Kasp_FHB_2DL* and *Kasp_FHB_3BL.*.

QTL	KASP	Primer	Sequence
*QFhb.haas-2DL*	*Kasp_FHB_2DL*	Flank sequence	5’-CAAGTGGCAACTTCTGGTTCCGCTGAGAGACTGACYAGAAAAGTGTCCCCCATGTGCGCCAGTGCCTACTC-3’
		FAM	GTTGGAGTGGTTGAAGTAGGA *ggacccgtcttcttgctctC*
		HEX	GTTGGAGGGTGAGTTGGGTAA *ggacccgtcttcttgctctT*
		Common	gcatgctgaactcccaacct
*QFhb.haas-3BL*	*Kasp_FHB_3BL*	Flank sequence	5’-AACAACCTCTTCAGTGGGACCCGTCTTCTTGCTCTYCGTCAACAGAATAGGTTGGGAGTTCAGCATGCCGC-3’
		FAM	GAAGGTGACCAAGTTCATGCT *gttccgctgagagactgacT*
		HEX	GAAGGTCGGAGTCAACGGATT *gttccgctgagagactgacC*
		Common	tgggtggtgggcctgagT

**Table 3:** Effects of *Kasp_FHB_2DL* and *Kasp_FHB_3BL* on FHB resistance in the natural population.

Marker name	QTL	Genotype	Number of lines	Phenotype	*P*-value
*Kasp_FHB_2DL*	*QFhb.haas-2DL*	AA	51	41.6	0.014*
a		GG	55	49.6	
*Kasp_FHB_3BL*	*QFhb.haas-3BL*	CC	55	40.2	0.008*
b		TT	48	52.1	

^a^AA is favorable allele, CC is unfavorable allele;

^b^CC is favorable allele, TT is unfavorable allele;

*Significant at *P* < 0.05.

**Figure 3:**
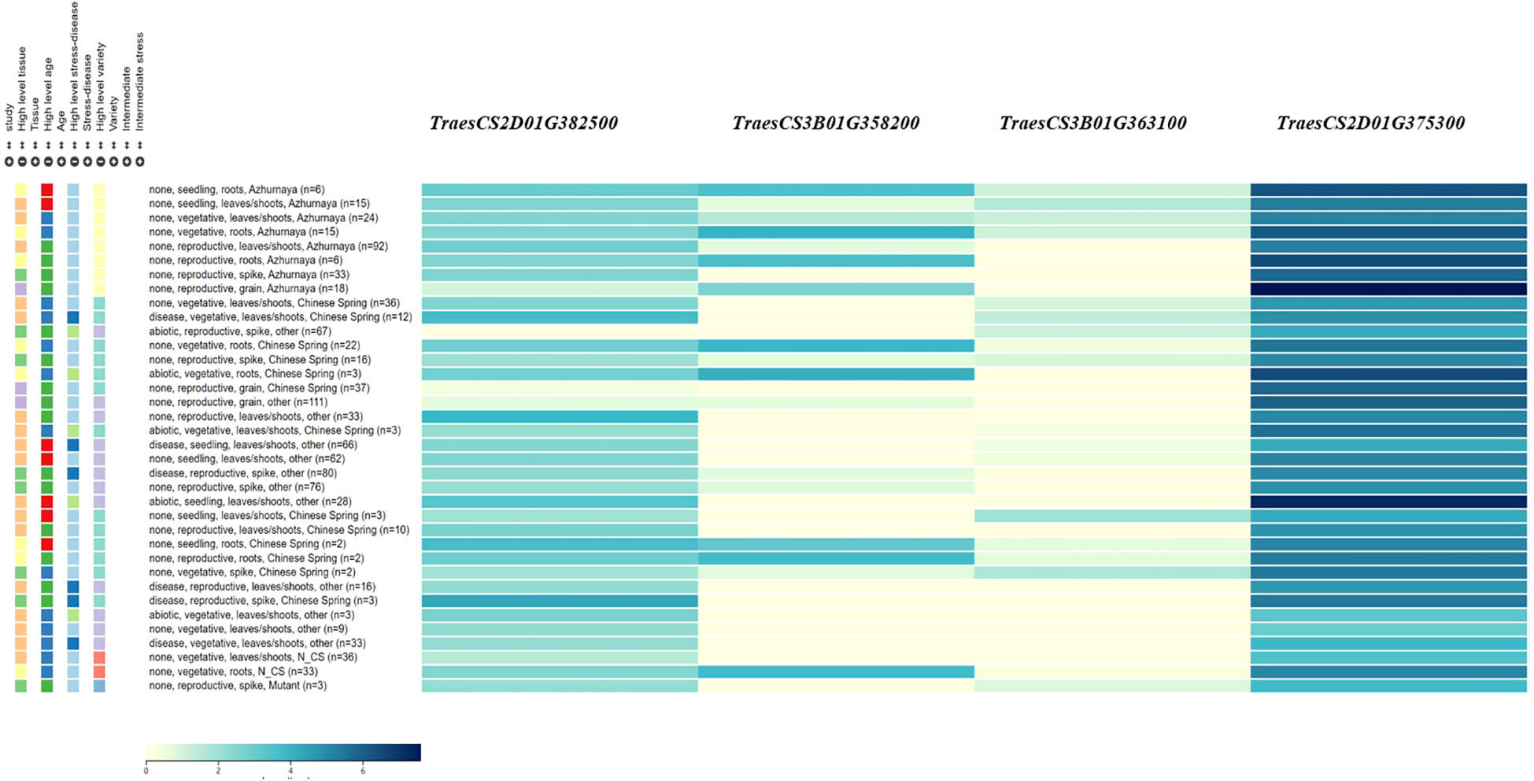
The expression patterns of the candidate gene identified in this study.

Four candidate genes were identified within the QTL regions and shown by qRT-PCR to have different expression levels between the two mapping population parents, primarily involved in plant hormone metabolism, cellulose synthesis, and the ubiquitin pathway (**Table 4** and **Figure 3**). These include *TraesCS2D01G375300* (encode the gibberellin 2-beta-dioxygenase), *TraesCS2D01G382500* (encode the histidine triad nucleotide binding protein), *TraesCS3B01G358200* (encode the serine/threonine-kinase WNK-like protein), *TraesCS3B01G363100* (encode the UDP-glycosyltransferase (**Supplementary Table S3**). All of the four candidate genes showed 1.4-3.9 folds higher expression in PS5/V975 compared to Sumai 3, suggesting their potential roles in FHB resistance mechanisms (**Supplementary Table S3**, **Supplementary Figure S1**).

**Table 4:** Candidate genes for FHB resistance identified in the PS5/V975/Sumai3 RIL population.

Candidate gene	QTL	Position (Mb)	Annotation
*TraesCS2D01G375300*	*QFhb.haas-2DL*	479.2	Gibberellin 2-beta-dioxygenase
*TraesCS2D01G382500*	*QFhb.haas-2DL*	486.4	Histidine triad nucleotide binding protein
*TraesCS3B01G358200*	*QFhb.haas-3BL*	571.0	Serine/Threonine-kinase protein
*TraesCS3B01G363100*	*QFhb.haas-3BL*	574.7	UDP-glycosyltransferase

## Discussion

Studies indicate that germplasm resources with FHB resistance are rare, and no immune materials have been identified, although several resistance sources have been found in hexaploid wheat, with the Chinese cultivar Sumai 3 and its derivatives being among the most prominent. In China, particularly in the Yellow and Huai River Valley, the incidence of FHB has increased over the past two decades, largely due to factors such as long-term maize-wheat rotation, straw retention, reduced tillage practices, and climate change (Zhu et al., 2018). Although agronomic and agrochemical measures can partially mitigate FHB, enhancing host resistance remains the most effective and sustainable strategy, and the development of tolerant or resistant cultivars is widely regarded as the most promising approach for controlling FHB. Despite progress in breeding for FHB resistance, conventional breeding techniques are insufficient for rapidly developing highly resistant wheat varieties, and the limited genetic diversity within the wheat gene pool poses substantial challenges. To address this, breeders have increasingly turned to genetic resources from wild relatives, and by creating SHW through crosses between modern durum wheat and *Aegilops tauschii*, new genetic resistance against FHB can be introduced into accessible germplasm for wheat breeding.

The main reason for selecting the single-floret inoculation method in this study lies in its precise targeting of Type II resistance (resistance to intra-spike pathogen spread) and high reproducibility in phenotypic assessment (Li et al., 2019a; Su et al., 2019). By injecting a quantified spore suspension of *Fusarium graminearum* into a single floret of the wheat spike, this method strictly controls inoculum dosage, infection site, and environmental disturbances (e.g., the impact of wind or humidity on spore dispersal). This precision ensures the comparability of the trait “restricting pathogen spread from the inoculated floret to adjacent florets or the rachis” across different wheat lines – a critical requirement for genetic mapping.

This accuracy is particularly vital for our study: the core function of well-characterized major FHB resistance genes (such as *Fhb1*) is to reduce disease severity by inhibiting hyphal growth within the spike, and the single-floret method directly quantifies pathogen spread rate-a phenotype that is functionally linked to *Fhb1* (Zhu et al., 2019; Hao et al., 2020). Thus, this approach allowed us to focus on “intra-spike spread resistance,” a trait of paramount importance for breeding, and the QTLs we identified (e.g., loci on chromosomes 2DL and 3BL) are more readily interpretable in the context of known resistance mechanisms. However, it is important to acknowledge that this targeted focus also limits our phenotypic data to a single dimension of resistance: we did not assess initial infection resistance (Type I) or comprehensive resistance under natural field conditions. While the single-floret method excels at dissecting Type II resistance, different inoculation techniques correspond to distinct resistance types, and their results are complementary. For example: Spray inoculation (uniformly spraying spore suspensions onto entire spikes) mimics the natural “airborne transmission-random infection” process of field pathogens, making it ideal for evaluating Type I resistance-the ability of a variety to prevent spore germination, penetration of floret scales, or colonization of stigmas. Grain spawn inoculation (burying infected wheat grains in soil) simulates the field infection cycle of pathogens that overwinter in crop residues, reflecting a variety’s performance under the complex “soil-borne inoculum - airborne spread - multiple infections” scenario-i.e., comprehensive field resistance. These methods could uncover QTLs not detected in our study. By focusing exclusively on Type II resistance, we may have missed loci associated with Type I resistance-yet these are critical for breeding “full-growth-stage resistant” varieties. For instance, a variety that blocks both “initial infection” and “intra-spike spread” will exhibit far stronger field resistance than one with only a single resistance type.

A single inoculation method can only capture partial dimensions of resistance (Wang et al., 2020). To build a more complete genetic model of FHB resistance, future studies must integrate multiple techniques: Combining single-floret inoculation (for Type II resistance) with spray inoculation (for Type I) will not only identify QTLs specific to each resistance type but also reveal their synergistic effects in comprehensive resistance. For example: A QTL significant only in spray inoculation likely functions in preventing initial infection. A QTL significant in both methods may mediate synergistic resistance to initial infection and intra-spike spread. Incorporating field data from grain spawn inoculation will validate QTL effectiveness under natural epidemic conditions—a non-negotiable step for translating laboratory-identified loci into practical breeding markers. In short, merging multiple methods will overcome the limitations of any single approach. It will not only reveal a more comprehensive genetic basis of resistance but also provide precise molecular targets for “polygenic pyramiding breeding”-e.g., stacking QTLs for Type I and Type II resistance to develop “dual-resistant” varieties that are both high-yielding and FHB-safe.

To date, nearly 100 FHB resistance QTLs or genes have been identified from common wheat and related species through linkage analysis or association mapping. In this study, we identified three loci: *QFhb.haas-2DL*, *QFhb.haas-3BS*, and *QFhb.haas-3BL*. The 3B chromosome is a gene-rich region for FHB resistance, with over 10 loci identified on 3B to date. Notably, most of these loci are located on the 3BS arm (e.g., *Fhb1* and *QFhb.caas-3BL*). Also, previous studies have identified several QTLs on the chromosome 3BL. Bourdoncle and Ohm (2003) reported a Type II resistance QTL linked with *Xgwm247* (826.2 Mb), while Zhu et al. (2020) identified an FHB resistance locus associated with *AX-110917097* (813.2 Mb) and *AX-110429468* (815.4 Mb). Additionally, Paillard et al. (2004) identified a locus for FHB resistance in Arina (*QFhs.fal-3BL*, 505.0 Mb, *Xcfa2134*). In this study, *QFhb.haas-3BL* was identified located in the genetic interval of 571.1-575.1 Mb and differs from these previously reported loci, suggesting that it may be a novel locus for FHB resistance. It is crucial to clarify that our study identifies *QFhb.haas-3BL* as a quantitative genetic locus, not a cloned gene. The claim of novelty is based on a physical position comparison with the IWGSC RefSeq v1.1, showing no overlap with previously reported loci on 3BL. To definitively confirm its novelty and function, a rigorous strategy is required: fine-mapping in a larger population to narrow the interval, followed by gene cloning and functional validation via transgenic complementation or gene editing. This will distinguish it from known genes and confirm its causal role in FHB resistance.

The 2D chromosome is another gene-rich region for FHB resistance. Over 10 loci have been reported on chromosome 2DL from various donors, including Wuhan 1 (130.8-577.4 Mb; Somers et al., 2003), Wangshuibai (513.1-728.6 Mb; Lin et al., 2006), CJ9306 (470.2-577.4 Mb; Jiang et al., 2007), Yangmai 158 (516.6-587.6 Mb; Yan et al., 2021; Zhang et al., 2021), Ji5265 (524.9-531.7 Mb; Li et al., 2022), Shanghai3/Catbird (130.8-577.4 Mb; Lu et al., 2013), and Soru#1 (571.2-574.4 Mb; He et al., 2016). The locus *QFhb.haas-2DL* (475.0-487.6 Mb) identified in this study, is closely associated with the locus from CJ9306 (470.2-577.4 Mb) (Jiang et al., 2007).

The incidence and severity of FHB are highly dependent on climatic conditions and the genotype of wheat varieties. Wheat resistance to FHB is primarily mediated through salicylic acid (SA) and jasmonic acid (JA) signaling pathways, which activate defense-related genes (Haile et al., 2020; Michel et al., 2021). Based on this knowledge, we identified putative candidate genes within the target QTL regions. Four candidate genes were selected, primarily involved in plant hormone metabolism, general metabolism, and immune response. Two candidate genes, *TraesCS2D01G375300* and *TraesCS2D01G382500* were identified for *QFhb.haas-2DL.* Of these, *TraesCS2D01G375300* encoded the gibberellin 2-beta-dioxygenase, which may enhance the signaling of defense hormones such as JA by regulating hormonal balance, thereby activating the resistance pathways against FHB (Ma et al., 2014; Guo et al., 2023). *TraesCS2D01G382500* encodes the histidine triad nucleotide-binding protein, which plays a significant role in regulating plant disease resistance (Wu et al., 2015). Overexpression of *Zm-HINT1* in *Arabidopsis thaliana* has been shown to enhance resistance to *Fusarium graminearum*. Additionally, *TraesCS3B01G358200* and *TraesCS3B01G363100*, located within *QFhb.haas-3BL*, encode a serine/threonine kinase WNK-like protein (Gupta et al., 2018; Xia et al., 2022) and a UDP-glycosyltransferase (He et al., 2018, 2020), respectively. Serine/threonine kinase WNK-like protein can regulate multiple signaling pathways through phosphorylation. In plants, such kinases may participate in modulating disease resistance-related signaling pathways, such as the JA or SA pathways, thereby enhancing the plant defense capabilities against pathogens. UDP-glycosyltransferases play a crucial role in wheat resistance to FHB by participating in processes such as secondary metabolism, toxin detoxification, and hormone signaling regulation through glycosylation.

The incorporation of the D genome from *Aegilops tauschii* into SHW has resulted in diverse populations with enhanced resistance or tolerance to various biotic and abiotic stresses, demonstrating significant advantages in resistance (Biselli et al., 2018; Ma et al., 2020). While durum wheat is generally susceptible to FHB, the introgression of resistance genes from hexaploid wheat has improved resistance in some durum wheat lines. Additionally, research has shown that the D genome, which is absent in durum wheat, plays a crucial role in FHB resistance. For instance, the introduction of the D genome from *A. tauschii* into SHW populations reduced disease severity by 18.3% compared to tetraploid counterparts (Szabo-Hever et al., 2018; Serajazari et al., 2023). In this study, *QFhb.haas-2DL*, originating from the D genome, further confirmed the pivotal role of the D genome in enhancing FHB resistance. These findings indicate that the incorporation of the D genome provides important genetic resources for breeding wheat with improved FHB resistance.

Although conventional breeding has made significant progress in improving FHB resistance in wheat, the selection process is time-consuming and often lacks precision. The occurrence of FHB in the field is heavily influenced by environmental factors, and the complexity and poor reproducibility of resistance phenotyping pose significant challenges to breeding efforts. To address these issues, MAS has emerged as a crucial tool for enhancing breeding efficiency. Among these, the KASP technique, known for its cost-effectiveness, flexibility, and high accuracy, has provided strong support for FHB resistance breeding. In this study, two molecular markers, *Kasp_FHB-2DL* and *Kasp_FHB-3BL*, were successfully developed based on SNP markers tightly linked to FHB resistance, offering reliable tools for resistance screening in breeding programs. The application of these markers not only accelerates the identification and selection of resistance genes but also effectively reduces breeding costs. Additionally, a group of varieties with favorable alleles, excellent FHB resistance, and desirable agronomic traits, such as Zhengmai 6, Hua 2566, Zhengmai 5 and Zhengmai 168, were identified as valuable parental materials for improving FHB resistance.

By integrating traditional breeding methods with modern molecular techniques, the efficiency and precision of FHB resistance breeding can be significantly enhanced, laying a solid foundation for developing high-yield, disease-resistant wheat varieties. Improving FHB resistance has become a primary goal in wheat breeding. In the future, further exploration of new resistance genes and the development of more efficient molecular markers will be key directions in FHB resistance breeding. Based on the above, we propose three recommendations for FHB resistance breeding: (1) In terms of resistance source utilization, select varieties with *Fhb1* and excellent agronomic traits, such as Sumai 3 and Yangmai 16, as parents, while paying attention to their winter or spring growth habits in practical applications. (2) In breeding, select high-yield, adaptable varieties as parents, perform 1–2 backcrosses with large populations, and use marker identification combined with FHB screening to develop new varieties with moderate FHB resistance and superior agronomic traits.(3) Gradually pyramid other FHB resistance loci to further enhance resistance levels.

While we developed KASP markers for MAS, its limitations must be acknowledged. When stacking multiple loci like *QFhb.haas-2DL* and *QFhb.haas-3BL*, challenges include epistatic interactions that may dampen additive effects, the logistical burden of multiplexing, and potential linkage drag from donor parents. Furthermore, MAS for a few major QTLs may not capture the full polygenic nature of FHB resistance, potentially overlooking minor-effect genes that contribute to comprehensive field resistance. MAS is most powerful when integrated with modern breeding platforms. Genomic Selection (GS) can model all major and minor QTLs, complementing MAS for polygenic traits. Haplotype-based selection can identify superior allelic combinations across breeding lines. Ultimately, once causal genes are validated, gene editing (e.g., CRISPR-Cas9) could precisely introduce favorable alleles into elite varieties, bypassing linkage drag. This synergistic approach accelerates the development of durable, high-yielding resistant cultivars. Pyramiding QFhb.haas-2DL and QFhb.haas-3BL is expected to yield additive resistance gains. Based on their individual PVEs (6.2-10.4%), stacking could potentially reduce the FHB index by an estimated 13-17%. However, biological limitations such as epistasis and genetic redundancy, alongside logistical hurdles in tracking and fixing multiple unlinked loci in large breeding populations, must be managed. This requires careful parent selection and large population sizes to break unfavorable linkages.

There are still many other limitations in our study, which pose challenges for the functional validation of genetic loci and their application in breeding. It is important to acknowledge the limitations of this study to contextualize its findings. Firstly, the resolution of our QTL mapping was constrained by the moderate density of the 50K SNP array, particularly in the D genome. This likely resulted in broad confidence intervals, complicating the precise identification of causal genes. Future fine-mapping with higher-density markers is warranted. Secondly, the genetic diversity captured in our biparental RIL population, derived from a cross between a single SHW line and Sumai 3, is inherently limited. The QTLs identified represent only the alleles segregating between these two parents, and their effects may be influenced by specific epistatic interactions within this genetic background. This affects the generalizability of our results, necessitating validation in more diverse genetic backgrounds to confirm their broad utility in breeding programs. Finally, while our multi-environment trials demonstrated QTL stability across four site-year combinations, the potential for genotype-by-environment (G×E) interactions requires further investigation. Our evaluation was conducted in a specific regional context, and different environmental conditions, such as those in other wheat-growing zones with distinct FHB pressure, might alter the expression and effectiveness of these QTLs. More extensive multi-environment testing is essential to fully assess their stability and optimal deployment strategies. In conclusion, addressing these limitations through fine-mapping, validation in diverse panels, and broader environmental testing will be crucial for effectively leveraging these QTLs in breeding for durable FHB resistance.

## Conclusion

This study highlights the potential of SHW as a valuable genetic resource for enhancing FHB resistance in common wheat. In this study, *QFhb.haas-2DL*, *QFhb.haas-3BS*, and *QFhb.haas-3BL* were identified for FHB resistance. In addition, the development and validation of two KASP markers, *Kasp_FHB_2DL* and *Kasp_FHB_3BL*, provide available molecular tools for the wheat FHB resistance breeding.

## Data Availability

The original contributions presented in the study are included in the article/**Supplementary Material**. Further inquiries can be directed to the corresponding authors.

## Supplementary material

Supplementary Figure 1 The qRT-PCR results for the candidate genes identified in this study.

Supplementary Table 1 Analysis of variance of FHB index in the PS5/V975/Sumai3 RIL population.

Supplementary Table 2 The genotypic and phenotypic data of FHB index for 108 wheat cultivars of KASP markers identified in this study.

Supplementary Table 3 The primers of the candidate genes identified in this study.
