# Supplementary figures and images for: Genome-wide linkage mapping of Fusarium head blight resistance in common wheat (Triticum aestivum L.)
Source: Front Plant Sci. 2025 Nov 10;16:1660303. doi: 10.3389/fpls.2025.1660303 (PMC12640948; doi:10.3389/fpls.2025.1660303)

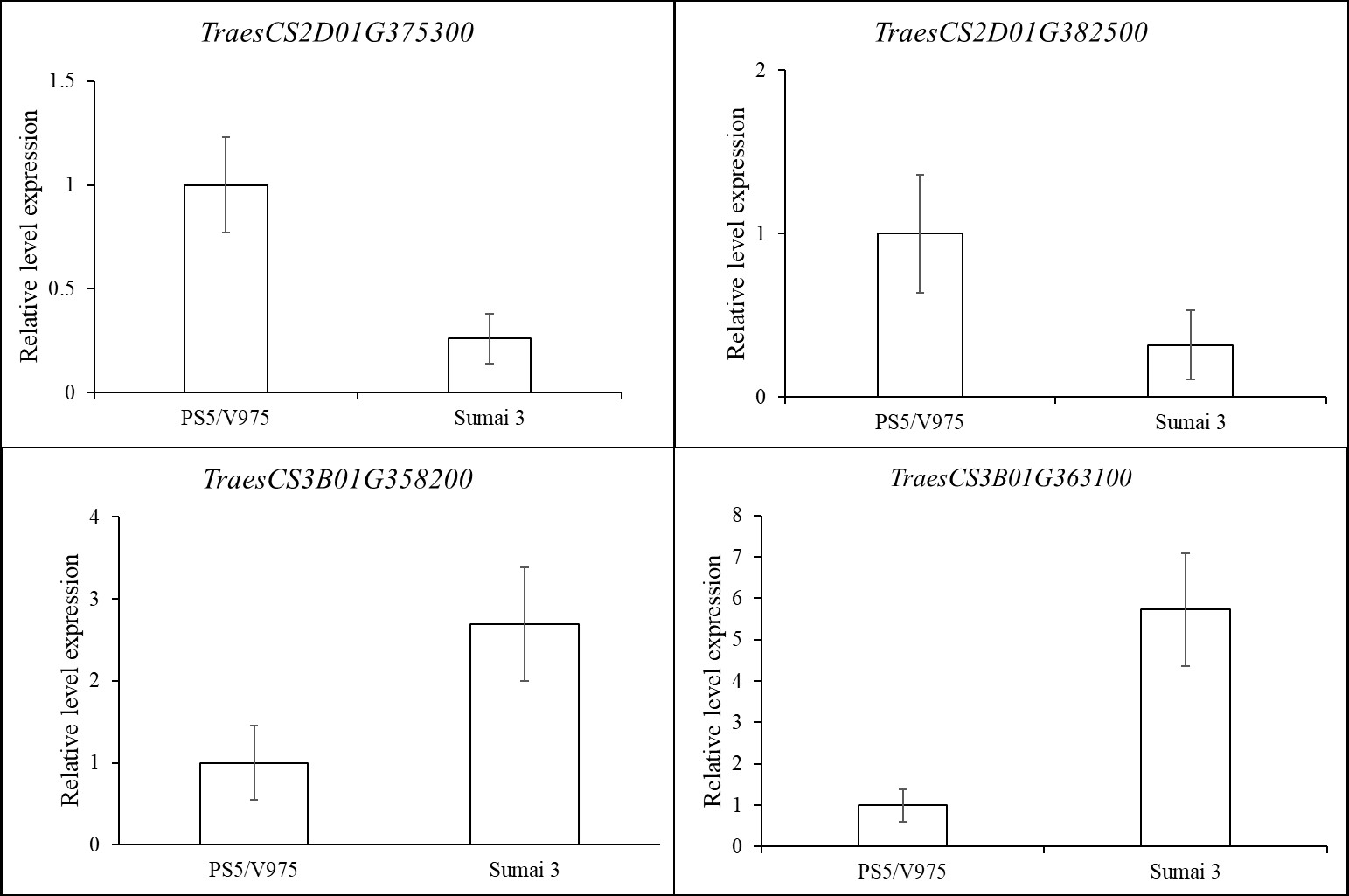

Supplement: Supplementary Figure 1 — The qRT-PCR results for the candidate genes identified in this study. [file Image1.jpg]
