# Supplementary material for: Genome-wide linkage mapping of Fusarium head blight resistance in common wheat (Triticum aestivum L.)
Source: Front Plant Sci. 2025 Nov 10;16:1660303. doi: 10.3389/fpls.2025.1660303 (PMC12640948; doi:10.3389/fpls.2025.1660303)
Supplement: Supplementary Table 1 — Analysis of variance of FHB index in the PS5/V975/Sumai3 RIL population. [file Table1.docx]

**Table S1** Analysis of variance of Fhb index in the PS5/V975/Sumai3 RIL population.

| **Source of variations** | **Df** | ***F-value*** |
| --- | --- | --- |
| Replicate (Environment) | 2 | 46.9** |
| Environment | 3 | 832.1** |
| Line | 214 | 22.9** |
| Line×Environment | 642 | 14.9** |
| Error | 1720 |  |
