# Supplementary material for: Genome-wide linkage mapping of Fusarium head blight resistance in common wheat (Triticum aestivum L.)
Source: Front Plant Sci. 2025 Nov 10;16:1660303. doi: 10.3389/fpls.2025.1660303 (PMC12640948; doi:10.3389/fpls.2025.1660303)
Supplement: Supplementary Table 2 — The genotypic and phenotypic data of FHB index for 108 wheat cultivars of KASP markers identified in this study. [file Table2.docx]

**Table S2** The genotypic and phenotypic data of Fhb index for 108 wheat cultivars of KASP markers identified in this study.

| **Number** | **Name** | ***Kasp_2DL_FhbR*** | ***Kasp_3BL_FhbR*** | **Fhb index (%)** |
| --- | --- | --- | --- | --- |
| 1 | Wanmai 52 | TT | CC | 45 |
| 2 | Wanmai 38 | TT | CC | 35 |
| 3 | Wanmai 50 | TT | TT | 40 |
| 4 | Kenong 9204 | CC | CC | 55 |
| 5 | Han 6172 | CC | TT | 60 |
| 6 | Kenong 199 | CC | TT | 70 |
| 7 | Jimai 38 | CC | TT | 65 |
| 8 | Shijiazhuang 8 | CC | TT | 70 |
| 9 | Heng 4422 | CC | TT | 50 |
| 10 | Hengguan 35 | CC | TT | 70 |
| 11 | Heng 136 | CC | TT | 60 |
| 12 | Yanzhan 4110 | CC | CC | 50 |
| 13 | Luohan 6 | TT | CC | 50 |
| 14 | Xinmai 16 | TT | CC | 55 |
| 15 | Ping'an 6 | TT | TT | 45 |
| 16 | Yumai 70-36 | TT | CC | 40 |
| 17 | Zhengmai 9694 | CC | CC | 55 |
| 18 | Zhengyu Mai 958 | CC | TC | 48 |
| 19 | Keda 9612 | CC | TT | 75 |
| 20 | Zhoumai 17 | TT | TT | 70 |
| 21 | Zhoumai 23 | CC | CC | 55 |
| 22 | Luomai 21 | TT | TC | 55 |
| 23 | Xiangmai 99 | CC | TT | 70 |
| 24 | Zhoumai 22 | CC | TT | 65 |
| 25 | Zhoumai 16 | TT | TT | 65 |
| 26 | Xinmai 18 | CC | TT | 60 |
| 27 | Xinmai 20 | TT | CC | 35 |
| 28 | Yumai 48 | TT | TT | 45 |
| 29 | Xinmai 22 | TT | CC | 40 |
| 30 | Luohan 7 | TT | TC | 55 |
| 31 | Wenmai 18 | CC | CC | 55 |
| 32 | Wenmai 19 | CC | TT | 70 |
| 33 | Xinmai 9817 | TT | CC | 40 |
| 34 | Xinmai 13 | CC | TT | 65 |
| 35 | Fanmai 5 | TT | TT | 60 |
| 36 | Zhengmai 004 | TT | CC | 55 |
| 37 | Yumai 69 | CC | CC | 65 |
| 38 | Zhengmai 98 | CC | CC | 50 |
| 39 | Zhengnong 17 | CC | TT | 75 |
| 40 | Yumai 70 | TC | TT | 40 |
| 41 | Kaimai 18 | TT | CC | 55 |
| 42 | Yumai 10 | TT | TT | 55 |
| 43 | Yumai 38 | CC | CC | 50 |
| 44 | Xinmai 19 | CC | TT | 50 |
| 45 | Zhongyu 10 | TT | CC | 55 |
| 46 | Xinmai 208 | TT | TT | 55 |
| 47 | Aikang 58 | CC | TT | 60 |
| 48 | Xumai 27 | CC | CC | 55 |
| 49 | Xumai 29 | TT | TT | 60 |
| 50 | Huaimai 20 | CC | CC | 35 |
| 51 | Huaimai 17 | CC | TT | 30 |
| 52 | Jimai 20 | TT | CC | 55 |
| 53 | Yannong 22 | CC | TT | 55 |
| 54 | Shannong 189 | CC | TT | 60 |
| 55 | Taishan 21 | TT | CC | 50 |
| 56 | Liangxing 99 | CC | TT | 55 |
| 57 | Yannong 19 | TT | TT | 50 |
| 58 | Jimai 21 | TT | CC | 50 |
| 59 | Tainong 18 | TT | CC | 55 |
| 60 | Shannong 16 | CC | CC | 45 |
| 61 | Hemiao 13 | CC | CC | 55 |
| 62 | Jimai 22 | CC | TT | 40 |
| 63 | Yannong 21 | CC | CC | 40 |
| 64 | Shannong 138 | CC | CC | 45 |
| 65 | Xiaoyan 22 | CC | TT | 55 |
| 66 | Xinong 979 | TT | TT | 55 |
| 67 | Xinong 3517 | TT | CC | 40 |
| 68 | Xinong 9871 | CC | TT | 65 |
| 69 | Shannong 757 | CC | CC | 40 |
| 70 | Shanmai 159 | TT | CC | 45 |
| 71 | Emai 596 | TT | TC | 20 |
| 72 | Emai 27 | TT | CC | 30 |
| 73 | E'en 1 | CC | CC | 25 |
| 74 | Emai 580 | TT | TT | 30 |
| 75 | E'en 6 | TT | TT | 35 |
| 76 | Emai 23 | CC | TT | 25 |
| 77 | Hua 2566 | TT | CC | 18 |
| 78 | Xiangmai 25 | TT | CC | 10 |
| 79 | Jingmai 103 | CC | TT | 40 |
| 80 | Yangmai 12 | CC | CC | 15 |
| 81 | Yangmai 158 | CC | CC | 15 |
| 82 | Yang 07-49 | TT | CC | 25 |
| 83 | Zhenmai 6 | TT | CC | 15 |
| 84 | Ningmai 16 | CC | CC | 38 |
| 85 | Zhenmai 5 | TT | CC | 20 |
| 86 | Yangmai 20 | TT | CC | 25 |
| 87 | Yangmai 15 | TT | TT | 40 |
| 88 | Ningmai 13 | CC | CC | 45 |
| 89 | Zhongma 895 | TT | CC | 5 |
| 90 | Ningmai 9 | TT | TT | 30 |
| 91 | Ningmai 8 | TT | CC | 35 |
| 92 | Zhenmai 168 | TT | CC | 30 |
| 93 | Yangmai 13 | CC | TT | 30 |
| 94 | Yang 07-15 | TT | CC | 45 |
| 95 | Yangmai 14 | CC | TT | 12 |
| 96 | Yangmai 16 | CC | CC | 15 |
| 97 | Yangmai 11 | TT | TT | 17 |
| 98 | Yangmai 17 | TC | TC | 18 |
| 99 | Chuanmai 42 | TT | TT | 40 |
| 100 | Mianmai 42 | CC | CC | 35 |
| 101 | Chuanmai 50 | CC | TT | 50 |
| 102 | Chuanmai 52 | TT | TT | 50 |
| 103 | Mianmai 37 | CC | CC | 55 |
| 104 | Lantian 13 | CC | CC | 28 |
| 105 | Lantian 12 | TT | CC | 38 |
| 106 | Ningchun 47 | TT | CC | 55 |
| 107 | Ningdong 11 | CC | CC | 40 |
| 108 | Ningchun 4 | CC | TT | 65 |
