# Supplementary material for: Genome-wide linkage mapping of Fusarium head blight resistance in common wheat (Triticum aestivum L.)
Source: Front Plant Sci. 2025 Nov 10;16:1660303. doi: 10.3389/fpls.2025.1660303 (PMC12640948; doi:10.3389/fpls.2025.1660303)
Supplement: Supplementary Table 3 — The primers of the candidate genes identified in this study. [file Table3.docx]

**Table S3** The primer of the candidate gene identified in this study.

| Candidate gene | Sequence-F (5’-3’) | Sequence-R (5’-3’) |
| --- | --- | --- |
| TraesCS2D01G375300 | AACAGGTACAAGAGCGTGGA | CTCTTGACATCGTCCTGCAC |
| TraesCS2D01G382500 | AAGAAGTCGTTGTGCTTCCG | GGATTGATGTCCTGGAACGC |
| TraesCS3B01G358200 | AGCTTCGTACGTGTCTCGAT | CCAGCCATCATGGAAGCAAA |
| TraesCS3B01G363100 | AAGCTGTCTTTGCTGGTGTG | CCAGTCCAACCTTCCAGTCT |
